# Hierarchical Structure Controls Nanomechanical Properties of Vimentin Intermediate Filaments

**DOI:** 10.1371/journal.pone.0007294

**Published:** 2009-10-06

**Authors:** Zhao Qin, Laurent Kreplak, Markus J. Buehler

**Affiliations:** 1 Laboratory for Atomistic and Molecular Mechanics, Department of Civil and Environmental Engineering, Massachusetts Institute of Technology, Cambridge, Massachusetts, United States of America; 2 Center for Materials Science and Engineering, Massachusetts Institute of Technology, Cambridge, Massachusetts, United States of America; 3 Department of Physics and Atmospheric Science, Dalhousie University, Halifax, Nova Scotia, Canada; 4 Center for Computational Engineering, Massachusetts Institute of Technology, Cambridge, Massachusetts, United States of America; George Mason University, United States of America

## Abstract

Intermediate filaments (IFs), in addition to microtubules and microfilaments, are one of the three major components of the cytoskeleton in eukaryotic cells, playing a vital role in mechanotransduction and in providing mechanical stability to cells. Despite the importance of IF mechanics for cell biology and cell mechanics, the structural basis for their mechanical properties remains unknown. Specifically, our understanding of fundamental filament properties, such as the basis for their great extensibility, stiffening properties, and their exceptional mechanical resilience remains limited. This has prevented us from answering fundamental structure-function relationship questions related to the biomechanical role of intermediate filaments, which is crucial to link structure and function in the protein material's biological context. Here we utilize an atomistic-level model of the human vimentin dimer and tetramer to study their response to mechanical tensile stress, and describe a detailed analysis of the mechanical properties and associated deformation mechanisms. We observe a transition from alpha-helices to beta-sheets with subsequent interdimer sliding under mechanical deformation, which has been inferred previously from experimental results. By upscaling our results we report, for the first time, a quantitative comparison to experimental results of IF nanomechanics, showing good agreement. Through the identification of links between structures and deformation mechanisms at distinct hierarchical levels, we show that the multi-scale structure of IFs is crucial for their characteristic mechanical properties, in particular their ability to undergo severe deformation of ≈300% strain without breaking, facilitated by a cascaded activation of a distinct deformation mechanisms operating at different levels. This process enables IFs to combine disparate properties such as mechanosensitivity, strength and deformability. Our results enable a new paradigm in studying biological and mechanical properties of IFs from an atomistic perspective, and lay the foundation to understanding how properties of individual protein molecules can have profound effects at larger length-scales.

## Introduction

Intermediate filaments (IFs), in addition to microtubules and microfilaments, are one of the three major components of the cytoskeleton in eukaryotic cells [1], [2], [3]. IFs are crucial in defining key biomechanical functions of cells such as cell migration, cell division and mechanotransduction, and have also been referred to as the “safety belts of cells” to prevent exceedingly large cell stretch [4], [5], [6]. Earlier studies focused on analyzing the mechanical signature of IFs have suggested that they are highly sensitive to applied forces at small levels, and that they can sustain extremely large deformation of up to 300% [3], [7], [8]. It has also been observed that due to severe stiffening, the tangent modulus of IFs increases manifold during deformation, a property that is believed to be crucial for providing mechanical stability to cells at large stretch. IFs are also found in the cell's nucleus in the form of lamin, where they form a dense mesh-like network providing mechanical integrity and biochemical functions at the cytoskeleton-chromatin interface [9], [10], [11]. IFs have also been associated with dozens of genetic diseases, where single point mutations and deletions lead to structural changes at different levels in the IFs organization. IF related diseases include muscle dystrophies, Alexander disease [12], epidermolysis bullosa simplex, as well as a broad class of disorders referred to as laminopathies (e.g. rapid aging disease progeria) [13].

**Figure 1:**
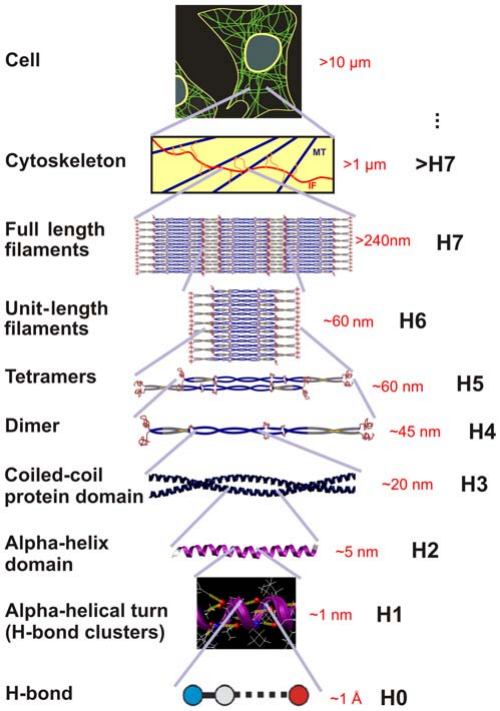
The hierarchical structure of intermediate filaments, from atomic to cellular scales. The figure shows relevant structural levels H0–H7. See Table 1 for deformation mechanisms associated with each hierarchical level.

**Figure 2:**
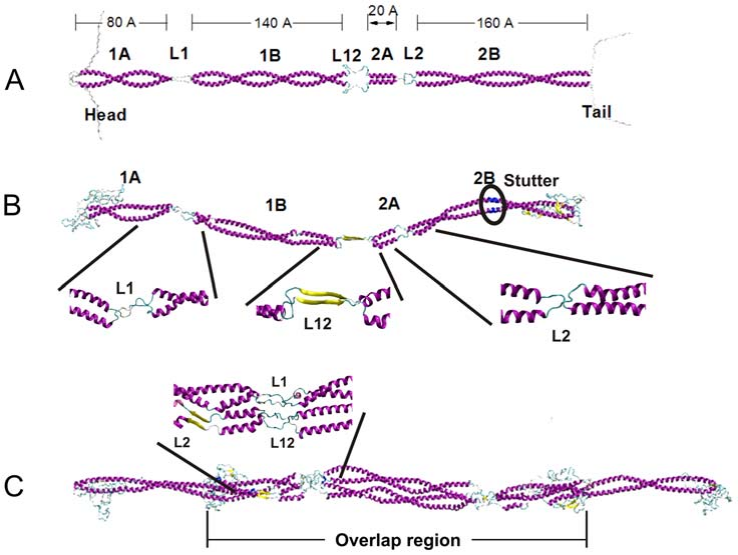
Molecular structures of vimentin dimer and tetramer (schematic and results of molecular simulation). Panel A: Schematic geometry of the dimer geometry, including labels identifying the various segments and linker domains. Panel B: Snapshot of the atomistic model of the dimer. The blue part denotes the stutter part around the stutter (amino acids 345–351). Panel C: Snapshot of the atomistic model of the tetramer. The blow-ups in panels B and C show detailed views of the molecular structure in the dimer and tetramer.

IFs form hierarchical structures as shown in Fig. 1, ranging from H-bonds, clusters of H-bonds in alpha-helical turns, alpha-helical proteins, dimers, tetramers, unit length filaments, full-length filaments to the cellular level. Each vimentin dimer contains 466 amino acids and forms the fundamental building block of IFs. Each dimer consists of four major structural segments linked in series in the sequence 1A, 1B, 2A and 2B, connected by linkers L1, L12 and L2 (Fig. 2A). Although there exists clear evidence that IFs play a key role in important physiological and pathological mechanisms, a complete atomistic-level molecular model of the basic constituents of this kind of protein material remains elusive. So far, only parts of two of the four segments, a section of the 1A and 2B domain of the vimentin dimer structure, have been crystallized and their atomic structure identified based on x-ray diffraction experiments [14], [15], [16], [17] (the structures are found in Protein Data Bank (PDB) entries 1gk4, 1gk6 and 1gk7). There are persistent experimental challenges in identifying the remaining parts of the vimentin IF structure. Since IFs are intrinsically disordered structures [18], x-ray diffraction studies on naturally occurring or recombinantly produced IF bundles do not provide sufficient data to produce a full atomistic model of IFs. Solid state nuclear magnetic resonance has been successfully utilized to derive atomistic models of amyloid fibrils [19]. However, amyloid peptides are much smaller than IF dimers, so that this approach may remain unsuccessful as well. Finally, cryo-electron tomography offers the promise of molecular-level imaging of single IFs, but the best tomograms are currently limited to a resolution around 5 nm [20].

Due to the lack of a structural model of IFs, little is known of the biological and physical material concepts of intermediate filaments. Specifically, our understanding of fundamental filament properties, such as the basis for their great extensibility, stiffening properties, and their exceptional mechanical resilience remains limited. This has prevented us from answering fundamental structure-function relationship questions related to the mechanical role of intermediate filaments, which is crucial to link structure and function in the protein material's biological context. The availability of a structural model of IFs is also the key to understand the mechanisms of IF related genetic diseases, where structural flaws cause major structural changes of biologically relevant properties. Here we utilize an atomistic-level model of the human vimentin dimer and tetramer to pursue a bottom-up nanomechanical analysis of the structure. In order to identify their nanomechanical properties at different levels in the material's hierarchical organization [21], we carry out a series of systematic molecular dynamics simulations to probe the response of the vimentin IF to mechanical tensile stretch. Specifically, the aim of our investigations is to elucidate the basis for their great extensibility, stiffening properties, and their exceptional mechanical resilience as well as associated molecular deformation mechanisms.

## Results

We apply a hierarchy of *in silico* techniques with different levels of accuracy, ranging from atomistic to coarse-grained representations, in order to enable a bottom-up multi-scale analysis of vimentin IFs at different hierarchical levels and time-scales. Atomistic level molecular dynamics simulations are carried out using the CHARMM19 all-atom energy function with an effective Gaussian model for the water solvent [22], [23], as well as by using explicit solvent simulations with the CHARMM force field and TIP3 water [24] implemented in NAMD [25] for validation of the effective Gaussian simulations. Additional validation simulations are carried out using the MARTINI force field [26], used to develop a coarse-grained representation of the proteins. Details are included in the Materials and Methods section.

### Molecular structure of vimentin dimer and tetramer

We first present the geometries and associated visualizations of the equilibrated geometries of both the dimer and the tetramer vimentin IF. We then continue with a detailed structural analysis of the resulting structures and compare with experimental measurements.

Figure 2B shows a snapshot of the molecular model of the vimentin dimer, displaying the characteristic segmented geometry with coiled-coil regions connected through linker domains. The total length of the dimer without the head and tail domain is ≈48.7 nm. This result is in agreement with experimental results where a range of 46–49 nm has been reported earlier [15], [27]. From dynamical analyses of the molecular dynamics trajectory of the dimer at 300 K, we observe that the linker domains represent soft, hinge-like structures that connect much stiffer coiled-coil segments (see Movie S1). This heterogeneous distribution of the bending stiffness along the molecule's axis strongly affects the filaments' motion by enhancing its mobility due an effectively reduced persistence length, which might be significant for IF self-assembly. Thereby, the linker domains act as hinges around which the molecular structure can rather easily bend and rotate.

Figure 2C shows a snapshot of the molecular model of the vimentin tetramer, consisting of two anti-parallel dimers [15]. The total length of the tetramer is ≈61.3 nm, which agrees with the length of unit length filaments observed in experiment (62 nm at pH 7.5 [27]). The overlap part of the tetramer has a length of ≈36 nm, where the experimental value is 30–36 nm [15], [27]. Segments 1A, 1B and 2A are fully contained in this part, but the 2B segments in the immediate vicinity of the two terminals are located completely outside. We find that the head segment of each dimer is coiled around the other dimer, increasing the contact surface area and thereby providing enhanced interdimer interactions. This structure is an alternative configuration to the sandwich model that has been discussed as a possible tetramer arrangement, where the head domain is located in between the two dimers [28]. Movie S2 shows the equilibrated structure of the vimentin tetramer, suggesting that the overlapped L1/L12 linker domains represent regions of locally soft bending stiffness similar to the dimer geometry. From an analysis of the movie it can be seen that compared with the dimer structure, the tetramer structure is notably more stiff due to its increased area moment of inertia, resulting in an effectively increased persistence length. Atomistic-level geometry PDB files of the dimer and tetramer can be found in Structure S1 (dimer) and Structure S2 (tetramer).

In both protein structures, the 1A segment contains 61 amino acids (residues 78–138), for which both phenylalanine and tyrosine residues are packed consecutively at the hydrophobic core positions near the segment center [29]. Therefore, the 1A segment is only marginally stable, which increases the mobility of the head domain during IF assembly as suggested in [17]. Segment 1B (residues 147–247) contains 101 amino acids with abundant hydrophobic residues distributed periodically around each alpha-helix. Therefore, the two chains generate a stable coiled-coil geometry, in agreement with results based on analytical ultracentrifugation [14]. Segment 2A (residues 264–282) is the shortest and the least flexible among all segments. It assumes the configuration of a nearly parallel alpha-helical bundle, in agreement with earlier suggestions [14]. Segment 2B with 115 amino acids (residues 291–405) features an alpha-helical coiled-coil geometry for the major segment, and a stutter region in the vicinity of residue 349. In the stutter region, the two alpha-helical chains unwind and give rise to a much longer coiled-coil pitch, resembling two parallel alpha-helices in that region (see Fig. 2B, region marked in blue). This regions is part of a segment that has already been crystallized, which also shows the formation of the stutter region [16], [30], confirming our predictions. The left end of the 1A segment connects to the head domain, while the right end of the 2B segment connects to the tail domain [31], where these two flexible domains are found to be poorly structured, in agreement with earlier suggestions [14], [17]. Linker L1 of 8 amino acids in length is among the softest regions in the structure, and facilitates the anti-parallel assembly of the dimers into larger-scale filaments, where these linkers align with the L12 domain in a regular pattern [32]. Linker L12 forms a beta-sheet structure in the dimer. An earlier sequence analysis has pointed out that the L12 domain region should form a small beta-sheet of two chains, with one face of the beta-sheet being apolar [32]. Our simulation confirms this hypothesis. Linker L2, which always features 8 amino acids in length for all IFs, results in a transition between two rod-like segments [32].

**Figure 3:**
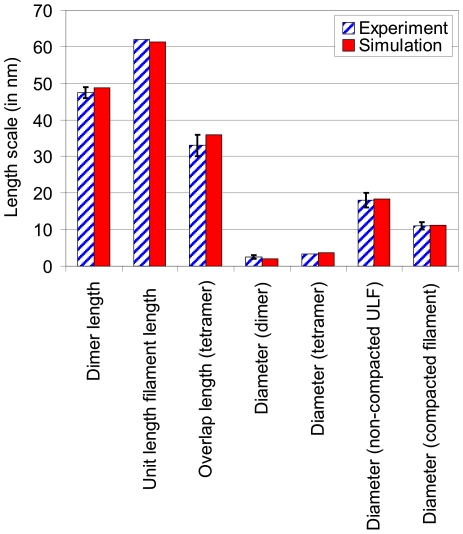
Comparison of structural properties of the IF dimer and tetramer between experiment and simulation (see also Table S1).

The average diameter of the cross section of the dimer is 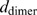 ≈2 nm, and the average diameter of the tetramer is measured to be 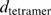 ≈3.6 nm, in close agreement with experimental measurements of 3.4 nm [15] (the small difference may be caused by the interaction with other dimers in larger filaments that contain many dimers, which may lead to increased compaction of the structure). Based on these geometric measurements we estimate that the diameter of a non-compacted unit length filament is 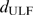 ≈18.3 nm, which is closely within the range measured in experiment, where values of 16–20 nm have been reported [33]. For compacted vimentin IFs we predict a diameter of 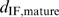 ≈11.2 nm, which is in excellent agreement with various experimental reports of values in the range of 10–12 nm [33], [34]. A summary of the structural comparison between simulation predictions and experimental results is shown in Figure 3 (and Table S1).

### Nanomechanical analysis of vimentin dimer and tetramer

**Figure 4:**
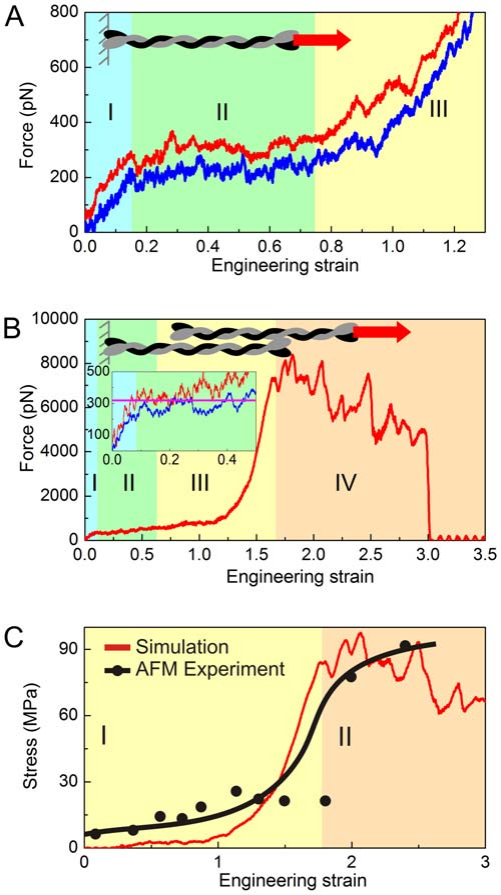
Force-strain relations for tensile deformation of the vimentin dimer, tetramer, as well as stress-strain relationship of full-length filaments (including comparison with AFM nanomechanics experiments). Panel A: Force-strain curve of the dimer (0.01 Å/ps and 0.1 Å/ps pulling speeds). The force-strain curve features three regimes: (I), The force increases linearly with strain until the rupture of the H-bonds and uncoiling of alpha-helices sets in. (II), A plateau of a constant force is found during which the unfolding process takes place. (III), The filament stiffens significantly due to stretching of the protein backbone. Panel B: Force-strain curve of the tetramer (0.1 Å/ps pulling speed; results for 0.01 Å/ps pulling speed are shown in the inlay for strains less than 50%). The pink bar in the inlay denotes the unfolding force for a dimer. The force-strain curve features four regimes: (I), The force increases linearly with strain until the rupture of the H-bonds and uncoiling of alpha-helices begins. (II), Plateau with approximately constant force, where the unfolding of 2B segment takes place. (III), Unfolding of the 1A, 1B and 2B segments. (IV), Interdimer sliding. Panel C: Stress-strain behavior of IFs, quantitative comparison between simulation and experiment. The black curve depicts experimental results of stretching single IFs (data taken from reference [8]). The two regimes (I) and (II) indicated by different shades refer to unfolding of alpha-helices and interdimer sliding between dimers beyond 175% strain, respectively.

We now proceed with a study of the nanomechanical properties of the dimer and tetramer structure by carrying out an engineering tensile test (for the pulling geometry, see inlays of Figs. 4A/B). Both structures are stretched until all helical domains are fully extended and/or failure due to intermolecular shear occurs. We begin with an analysis of the mechanical behavior of the dimer. Figure 4A shows two characteristic force-strain curves for two pulling speeds (0.01 Å/ps and 0.1 Å/ps). The simulations reveal three distinct regimes of deformation. In the first regime (I), the pulling force increases linearly with strain until it reaches an angular point, where a dramatic change in the slope emerges. In the second regime (II), the force-strain curve resembles a plateau regime where the pulling force remains almost constant. It is found that unfolding of all alpha-helical domains occurs in this regime (II). In the third regime (III), the stretching force increases rapidly as the strain increases. This is caused by pulling the unfolded polypeptide backbone of the protein, where the stretching of covalent bonds leads to a much increased stiffness. The overall characteristics of the force-strain curves of the dimer share similarities with stretching of individual alpha-helices [35], [36] as well as with myosin coiled-coil structures [37], which agrees with the fact that most parts of the dimer are composed of coiled-coils (see Fig. 2B). Regime (II) represents the main unfolding regime, where the average force in this regime approximates the protein's unfolding force.

We continue with an analysis of the tetramer mechanical properties. Figure 4B shows the force-strain curves of the tetramer. The simulations reveal four distinct regimes. In the first regime (I), the pulling force increases linearly with the strain until it reaches the angular point. In the second regime (II), the force-strain curve forms a plateau, where the pulling force keeps almost constant, which is similar to the case of the deformation properties of the dimer. From simulation snapshots, we find that the 2B segments unfold first, starting from the stutter location, then expanding throughout the entire segment. In the third regime (III), we observe a significant transition in the force-strain behavior. During this transition, the pulling force changes from the low-force plateau (<1 nN) to a peak value of 7.3 nN, marking the onset of the final regime (IV). Beyond the peak value, the force level in regime (IV) begins to decrease. The force plateau observed in Figs. 4A and B relates to the stepwise unfolding of the coiled-coil segments. We observe that at the end of the plateau regime, the dimer and tetramer behave differently. Once the dimer is completely uncoiled, the pulling force abruptly increases due to the stretching of the protein backbone. Whereas for the tetramer, the overlapped part in center begins to uncoil, and the pulling force approaches a much broader transition region, where the structure becomes significantly stiffer and eventual fails by interdimer sliding. The two dimers are completely detached at ≈300% strain (Fig. 4B). The comparison between the dimer and tetramer reveals a significant difference in the mechanical behavior.

### Comparison with experiment

**Figure 5:**
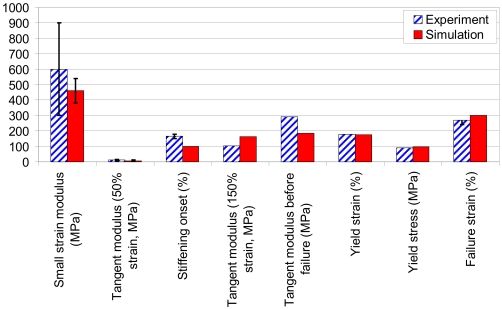
Comparison of mechanical properties of IF filaments between experiment and simulation (based on data shown in Table S2).

We present a quantitative comparison with AFM experiments of single IF filaments [8]. The comparison between simulation and experiment is shown in Fig. 4C, showing overall good agreement between simulation and experiment. Specifically, the Young's modulus for small deformation, calculated from the pre-angular point regime shown in Fig. 4A (where the dimer is deformed homogeneously), results in values in the range of ≈380–540 MPa. This is in close agreement with experimental results from corresponding vimentin IF bending experiments, which show moduli in the range of 300–900 MPa [38]. This agreement supports the notion that IFs should be considered as a continuous body in the very small-deformation regime where no unfolding or sliding occurs. The resulting maximum stress for the filament is predicted to be ≈97 MPa (found at ≈205% strain) from simulation (Fig. 4C), close to experimental measurements of 90 MPa at similar levels of strain [8]. The transitions of the stress-strain behavior and the maximum strength in the two methods show overall good agreement. However, in the lower strain regime before transition, the experimental stress is higher, possibly caused by effect of sliding of the filament on the surface during stretching. From the analysis of the stress-strain behavior, we extract the tangent modulus for the full length filament to be ≈3..12 MPa below 50% strain. This modulus is close to experimental results, where a range of ≈7..15 MPa was reported [8], [34]. We note that the initial tangent Young's moduli found here are significantly smaller than typical values reported for α-keratins (1.8 GPa), microtubules (0.9 GPa) [39] and F-actin (2.6 GPa) [40], confirming the characteristic properties of IFs. In both simulation and experiment, the tangent modulus increases by a factor of ≈20 once 150% strain is reached, leading to severe stiffening. The tangent modulus in simulation approaches ≈185 MPa before failure, similar to experimental results. Possible explanations for the discrepancies between experiment and simulation include the presence of defects, structural irregularities, and the fact that much longer filaments are considered in experiment, whereas the small system considered in our simulations is perfect without any structural flaws. A summary of the nanomechanical properties and a comparison between simulation and experiment is shown in Figure 5 (see also Table S2).

### Multi-scale deformation and failure mechanisms

**Figure 6:**
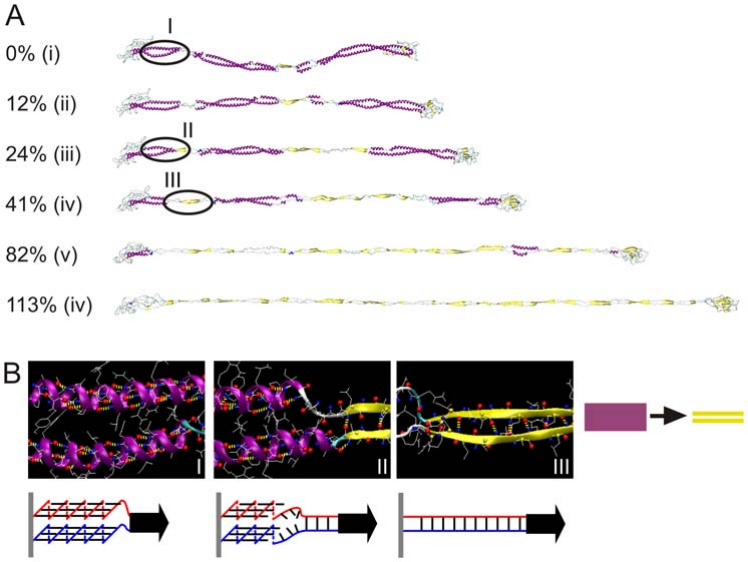
Simulation snapshots and structural analysis during pulling of a dimer. Panel A displays snapshots as the applied tensile strain is increased up to 113%. Panel B depicts snapshots of the right part of the 1A segment as the *α-β* transition occurs (frames I to III correspond to those highlighted in panel A). The schematics in the lower part describe the atomistic details of the *α-β* transition mechanism.

Our simulations of mechanical deformation across several orders of magnitudes of strain until failure occurs enable us to carry out a detailed mechanistic and structural analysis of deformation mechanisms of both the dimer and tetramer. We begin by analyzing cartoon visualizations of the filament structures at different levels of applied strain. Figure 6A displays the structural deformation during the stretching simulation of the dimer. Once tensile load is applied, the four segments align in the pulling direction, and the coiled-coil protein domains are stretched slightly to mediate the elongation. No rupture or domain unfolding is observed until the applied strain is larger than 12%, where the 2A segment begins to uncoil first. The uncoiling of alpha-helical structures occurs simultaneously with the formation of beta-sheets, a phenomenon referred to as *α-β* transition. The pulling force causes the two uncoiled chains to come sufficiently close to form a beta-sheet protein domain. Figure 6B shows atomistic-level details of the α-β transition process, illustrating the mechanisms by which beta-sheet are formed due to uncoiling of pairs of alpha-helical domains (results shown for the right part of the 1A segment). The *α-β* transition as observed here has also been observed in experiment, where it was shown that the secondary structure of double-stranded coiled coil proteins gradually transform into beta-sheets under applied tensile load [34], [41], [42].

**Figure 7:**
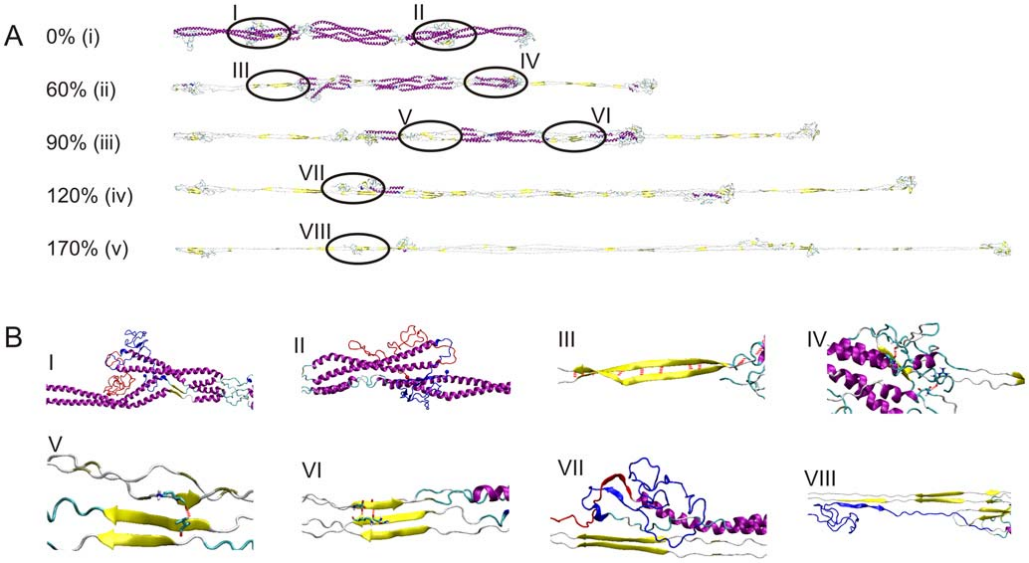
Simulation snapshots and structural analysis during pulling of the tetramer. Panel A displays snapshots as the applied tensile strain is increased up to 170%. The *α-β* transition leads to significant thinning of the filament diameter (compare snapshot (i) with snapshot (v)). Panel B displays blow-ups of detailed atomic structures of the snapshots marked (I) to (VIII) in panel A.

Figure 7A shows the structural characteristics of the tetramer under tensile deformation, showing molecular structure snapshots as the tetramer is being stretched. As the applied strain increases to 20%, the first part to unfold is the free-standing 2B segment (the *α-β* transition is also observed here). We find that at low strains there is no unfolding in the overlapped part, because the head part of each dimer forms H-bonds with the other dimer. Therefore, the two dimers are strongly connected in the head-coiled-coil overlap region. Once strain is increased to more than 60%, unfolding of the 1A segment in each of the two dimers begins, accompanied by beta-sheet formation. The anti-parallel beta-sheets formed by individual coiled-coil uncoiling processes interact with each other to form larger beta-sheet crystals as shown in Figs. 7B (see blow-ups). The three segments 1A, 1B, 2A begin to unfold sequentially between ≈60% and ≈100% strain. Unraveling of the 1A segment, the first to rupture at ≈60% strain, stops at ≈170% strain, requiring the largest applied strain among all three domains.

**Figure 8:**
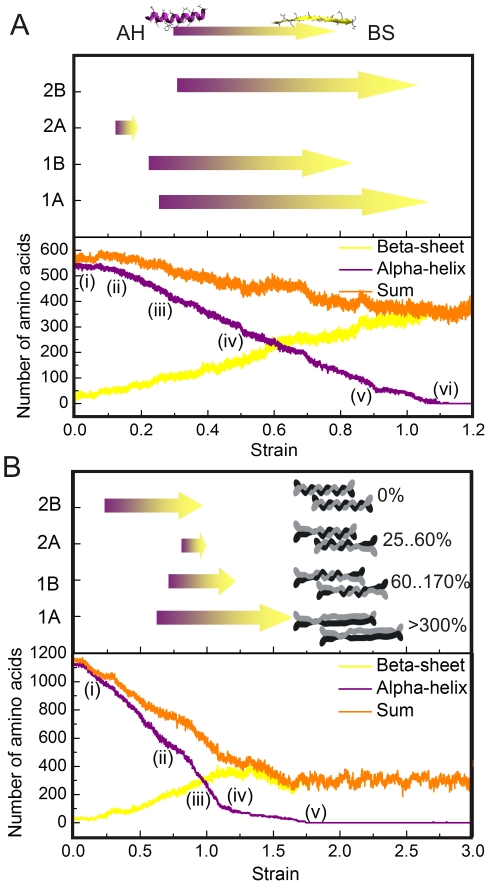
Geometry-strain maps for the dimer (panel A) and tetramer (panel B) structures. The lower part of the plots shows the number of amino acids associated with alpha-helical structure (purple) or beta-sheet structure (yellow), and the sum for these two known structures (orange). The arrows shown in the upper part of the plot denote processes of *α-β* transition for each segment. Each point marked corresponds to a snapshot with the same labels as shown in Figs. 6 and 7. The inlay in panel B shows schematics of the deformation mechanisms of the IF tetramer at various levels of strain.

Further structural analysis is shown in Fig. 8, where we plot the number of amino acids associated with alpha-helical and beta-sheet secondary structures as a function of the applied tensile strain in a geometry-strain map. We first focus on the dimer structure. The results shown in Fig. 8A confirm that the number of amino acids in the alpha-helical state is approximately constant for strains smaller than 12%, which demonstrates that the structure is intact. The reason why the 2A segment ruptures first is that while the other three segments are coiled-coils, the 2A segment is essentially a parallel alpha-helical bundle, which has a lower mechanical stability against tensile stretch as pointed out earlier [14], [21]. Once the 2A segment is fully uncoiled at ≈19% strain, the other segments 1B, 1A and 2B begin to uncoil sequentially. Furthermore, the geometry-strain map shown in Fig. 8A (upper part) confirms that the segments do not unfold simultaneously, but rather in a sequential process. The observed sequence with unfolding in the order 2A→1B→1A→2B is repeatable in different simulations with different pulling rates. As depicted in Fig. 8A, while the number of amino acids associated with alpha-helices decreases from ≈520 to 0 between 12% and 110% strain, the number of amino acids associated with beta-sheets increases from ≈10 to 370, supporting the notion that most amino acids in the dimer under pulling turn from alpha-helical dominant structures to beta-sheet structures (the ≈10 beta-sheet associated amino acids in the beginning correspond to the short beta-strand found in the L12 linker).

The behavior of the tetramer is more complex. As shown in Fig. 8B, here the 2A domain unfolds fastest, followed by the 1B domain. The unfolding sequence with unfolding in the order 2B→1A→1B→2A is repeatable in different simulations. Notably, the unfolding sequence in the tetramer is reversed when comparing it with the dimer. Once the 1A segment has completed the *α-β* transition, the structure is invariant beyond this region, as shown in Fig. 8B. It is also noted that unlike as in the case of stretching the dimer, the sum of the number of amino acids in alpha-helix and beta-sheet conformation in the fully stretched structure (≈320) is much smaller than its initial value (≈1130). This is due to the fact that the beta-sheets observed in the stretched tetramer are composed of discrete short beta-structured segments with a characteristic length of ≈28 Å (corresponding to 7 amino acids in length, with roughly 7 H-bonds each), while for stretched dimers the average beta-sheet length is ≈45 Å. Interdimer sliding sets in at strains in excess of 180%, which is mediated by the initiation of rupture of the small beta-sheet protein crystals.

**Figure 9:**
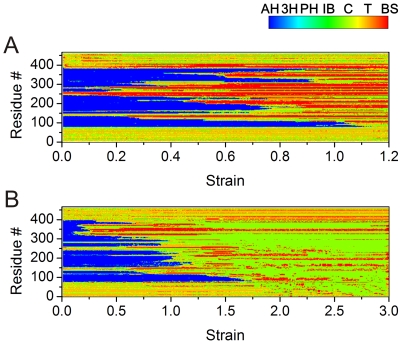
Structural spectrum of the vimentin dimer and tetramer, as a function of strain. The color bar indicates the structural character is separated into seven parts and corresponds to an α-helix (AH), 3–10 helix (3H), phi-helix (PH), isolated-bridge (IB), coil (C), turn (T) and a β-sheet (BS). Panel A: The spectrum of the structural transition of the vimentin dimer. The plot shows the structural character of each amino acid of a monomer (with 466 amino acids in total) in the vimentin dimer during the entire stretching process (due to the symmetric arrangement of the other monomer they show a same behavior, and is thus not shown). Panel B: The spectrum of the structural transition of the vimentin tetramer. The plot shows the structural character of each amino acid of a monomer (with 466 amino acids in total) in the vimentin tetramer during the entire stretching process (due to the symmetric and antisymmetric arrangement of the other three monomers, they show the same behavior, and are thus not shown). The analysis shows the gradual transition from an alpha-helix dominated structure (with unstructured and beta-sheet based linker/head/tail domains) towards a beta-sheet rich structure. In the case of the vimentin tetramer, the onset of sliding at ≈170% strain induces a reduction of the beta-sheet content as the strain is increased. The beta-sheets form many short segments in the overlapped region beyond this level of strain, leading to significant oscillations of the stretching force as shown in regime (IV) of Fig. 4B.

Figure 9 shows the structural spectrum of the vimentin dimer (Fig. 9A) and tetramer (Fig. 9B), as a function of strain, providing details into the structural transition of the protein as the strain is increased. The plot shows the structural character associated with each of the 466 amino acids, revealing the spatial distribution of the structural changes during deformation. The beta-sheets form a large number of relatively short segments in the overlapped region, leading to significant oscillations of the stretching force as shown in regime (IV) of Fig. 4B.

**Figure 10:**
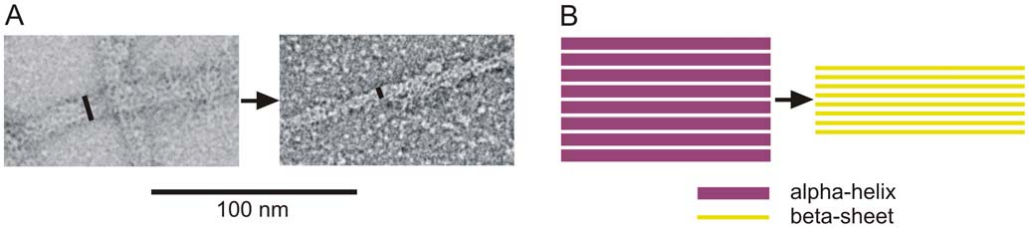
Experimental evidence for interdimer sliding and subsequent filament thinning. Panel A: Electron microscopy picture of a mutant IF desmin DesA360P filament before and after shearing (adapted from reference [43]). Significant thinning of the filament diameter of ≈55% seen in these experimental images suggests that *α-β* transitions and interfilament sliding occur, in agreement with simulation results (the diameter changes from ≈12 nm to ≈5.5 nm). Panel B: Schematic of the deformation process, illustrating the thinning of the filament due to formation of beta-sheets.

The deformation of IFs, involving the *α-β* transition and subsequent sliding suggests that significant filament thinning should occur. Indeed, this behavior has been observed experimentally in atomic force microscopy and electron microscopy studies of desmin IFs [8], [43] as shown in Fig. 10, in qualitative agreement with our predictions. Whereas the sliding of dimers or tetramers within a single filament has been proposed as a mechanism to explain the superelasticity of IFs, it was never directly observed at the single filament level. The only experimental evidence for sliding is based on a small angle x-ray diffraction study of stretched hard alpha-keratin fibers [44].

### Hierarchical structure is crucial to define mechanical properties

The hierarchical structure of IFs is crucial to achieve their characteristic mechanical properties, as it enables the cascaded activation of a different deformation mechanism, each at a specific level of tensile strain, to accommodate substantial mechanical deformation. Specifically, this cascaded activation of mechanisms at multiple levels in the structural makeup of IFs explains the remarkable behavior of IFs that renders them capable to withstanding extreme deformation and large loads. Thereby, the specific sequence of alpha-helix unfolding (providing great sensitivity to mechanical deformation, as shown in Figs. 8 and 9), subsequent *α-β* transition and interdimer sliding via beta-sheet crystal rupture ensures the mechanical function of IFs under a wide range of strains. The key to enable these mechanisms is the existence of specific structures at each hierarchical level.

We discuss here the detailed role of each level of hierarchy in the filament. The unfolding of alpha-helical domains, facilitated by repeated breaking of serially arranged clusters of H-bonds (see Figs. 6 and 7) absorbs stretching energy while keeping the other structures intact. Thereby, the grouping of H-bonds into clusters of 3–4 in each alpha-helical turn has been shown to be crucial in order to yield maximum mechanical strength against rupture, and contributes to the enhanced stability of alpha-helical turns [35]. The coiled arrangement of the polypeptide into turns provides the structural basis for increased “hidden length” of the backbone chain, which is being released when rupture occurs. This, together with the serial arrangement of multiple turns into long alpha-helical domains is the structural basis to enable their ability to stretch more than 140% at relatively low force levels on the order of hundreds of pN. The arrangement of two alpha-helices into coiled-coil domains provides increased strength and additional deformability to increase the overall mechanical stretchability in excess of 150% [45]. During the uncoiling of alpha-helical and coiled-coil structures, an *α-β* transition occurs. This is facilitated by allowing the uncoiled chains to connect via H-bonds, and thereby to form beta-strands (Fig. 6B), assembled into small clusters with several H-bonds each. These beta-shear topologies are highly efficient in resisting tensile force and thus control the mechanical behavior in the regime of large deformation at strain levels in excess of 150% (see Figs. 8B and 9B) [46]. The beta-sheet shear topologies also facilitate a significant increase of the tangent modulus as they provide sufficient force resistance to induce stretching of the polypeptide backbone. Once the applied force exceeds a critical value at 175% strain, interprotein sliding sets in as beta-sheets begin to rupture in a stick-slip like fashion, resulting in a force plateau with relatively large forces that continues up to 300% strain when the proteins detach and catastrophic failure occurs. Altogether, these mechanisms explain how IFs can sustain 300% deformation without catastrophic failure, while providing a continuously increasing mechanical stiffening as the tensile deformation is increased.

Table 1 summarizes the function of each hierarchical level in vimentin IFs in defining their mechanical properties. Overall, this analysis suggests that the hierarchical makeup of IFs is crucial in defining their characteristic biomechanical properties.

**Table 1:** Role and mechanism of individual levels of structural hierarchies in IFs (see Fig. 1 for schematic of structure and labeling of hierarchical levels).

Hierarchy level H*n*	Description	Key mechanism(s)
H0	Level of chemistry; intrabackbone H-bond	H-bonds form at moderate temperatures; Drive formation of alpha-helices
H1	Alpha-helical turn defined by cluster of 3–4 H-bonds	Clusters of 3–4 H-bonds provide maximum mechanical strength at minimal material cost [35]; Coiled geometry of polypeptide provides significant “hidden length”
H2	Alpha-helix domain	Linear array of alpha-helical turns enables large extensibility of >150% strain via repeated rupture of turns [35], [36]
H3	Coiled-coil protein domain	Increased stability and mechanical resistance [45]; Additional extensibility through coiled geometry [37]
H4	Dimer	Combination of several coiled-coil domains connected via linkers facilitates controlled unfolding segments (Fig. 6); Linkers form hinge-like structures (Fig. 2B)
H5	Tetramer	Staggered geometry provides basis for α-β transition with increased resistance to interfilament shear through formation of beta-shear topologies; Sustain stretching up to ≈300% at large force levels (Fig. 7)
H6	Unit length filament	Assembly intermediate (intermediate state between tetramers and full length IF filaments)
H7	Full length IF filament	Axially assembled from ULFs. Filaments with great stretchability, stiffening and superelastic properties (Fig. 4C)
>H7	Cytoskeleton, cell	Not explored here

The cascaded activation of mechanisms at multiple hierarchical levels is the key to explain the unique properties of IFs, and illustrates that each level of hierarchy contributes an important element to the overall properties at the filament level. Thereby, the structure at each level controls the associated mechanism, and the aggregate effect of all mechanisms explains the characteristic mechanical properties of IFs.

## Discussion

Here we developed an atomistic model of human vimentin dimer and tetramer IFs, validated against key structural parameter identified in experiment (Fig. 3). We carried out molecular dynamics simulations to measure their response to mechanical stress. To the best of our knowledge, this is the first nanomechanical analysis of the vimentin dimer and tetramer at atomistic and molecular resolution. Our analysis provides detailed insight into the molecular-level deformation mechanisms, and enabled us to link the force-strain behavior (Fig. 4) to geometric changes in the structural makeup of the protein at different hierarchical levels (Figs. 6 and 7 and Table 1). Our results are validated against experimental studies of the mechanical properties of IFs (Fig. 4C and 5). The overall good agreement between experiment and simulation suggests that our model captures the key structural and mechanical features of the vimentin IF. Specifically, the tetramer molecular simulation study highlights the two molecular ingredients elementary for IF tensile properties, an alpha-helix to beta-sheet transition, and a sliding process that begins at large deformation.

The most salient feature of vimentin and other IFs is that the filament is rather compliant at low strain, whereas it becomes much stiffer at high strain, as confirmed in the analysis shown in Fig. 4. We find that there is no sliding at small strain, suggesting that IFs should be considered as a continuous body at small strains. This is confirmed by the fact that the predictions put forth based on the results shown in Fig. 4A agree closely with experimental modulus measurements in bending. We find that large deformation of both the dimer and the tetramer is accompanied by an *α-β* transition, a phenomenon that has been observed for other long coiled-coil protein filaments such as α-keratin IF fibers [39], [41], [42], myosin [37] and IF hagfish slime threads [6], [34]. Our results further suggest that the hierarchical makeup of IFs is elementary in defining their characteristic mechanical properties through a cascaded activation of deformation mechanisms, each associated with a specific level of filament strain as shown in Table 1. This process, likely evolutionarily driven, combines disparate properties such as strength and superelasticity (the ability to tolerate large deformation without breaking). At larger levels, these properties may play a role in the protection of the structure and topology of the cell's cytoplasm under large deformation and at large forces. Furthermore, the characteristic sequence of segment unfolding as a function of different levels of strain (see e.g. Fig. 8) may be an important element in mechanotransduction, as each unfolded segment could perhaps facilitate the binding of specific signaling proteins, providing a means to enable biochemical signaling of different levels of stretch.

The assumptions made in the design of the computational approach used here are based on using methods that allow for the development of the most appropriate simulation model, given constraints such as simulation size, computational resources, and requirements based on the particular biophysics of the problem. Future work could be focused on further refinement of the structural model reported here, for example by using higher fidelity atomistic force field models, or perhaps by using methods that facilitate the enhanced sampling of structural configurations. However, due to the large size of the protein filaments considered here, the computational expense of such simulations can be significant, in particular if long time-scales are considered.

**Figure 11:**
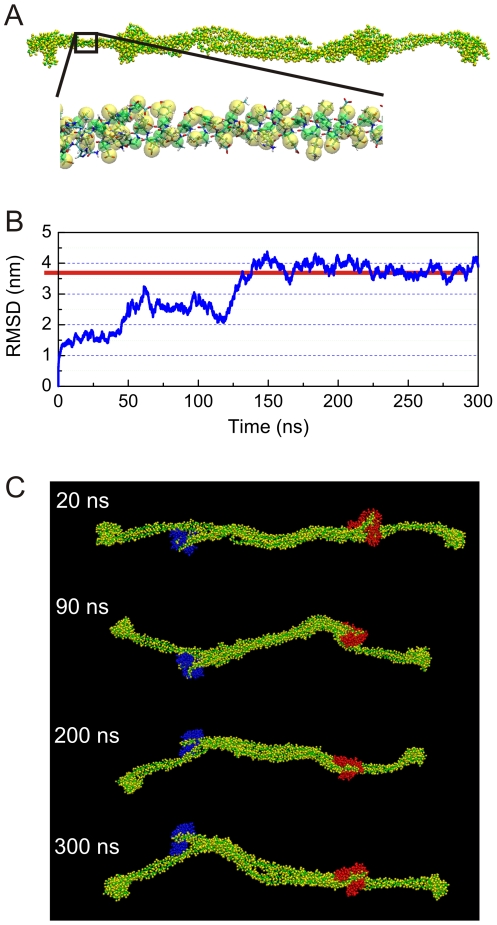
Coarse-grained representation of the vimentin tetramer (in explicit water) and structural stability of the vimentin tetramer structure at time-scales of hundreds of nanoseconds. By replacing the full atomistic representation with a bead model (where several beads reflect one amino acid), much longer time-scales can be reached. Panel A shows the coarse-grained representation of the vimentin tetramer (water molecules not shown for clarity). Panel B depicts the root mean square displacement function as a function of time, showing structural stability of the predicted structure. The root mean square displacement function provides a measure for how much a structure changes relative to the initial geometry. Panel C shows snapshots during the coarse-grained equilibration, indicating the head domains in a different color.

The use of other methods, such as replica exchange simulations or coarse-grained approaches could be explored to provide an additional comparison with the results obtained here, for example to reach a better sampling of structural configurations at long time-scales. Here we report a first simulation of the vimentin tetramer using a coarse-grained model with explicit water, as shown in Fig. 11A, where the initial geometry is based on the result of the full atomistic equilibration (structure shown in Fig. 2C). This coarse-grained model enables us to reach time-scales of hundreds of nanoseconds to microseconds. An analysis of the structural evolution of the tetramer in this coarse-grained representation provides further evidence for the stability of the predicted structure at long time-scales of hundreds of nanoseconds, as confirmed in the analysis shown in Fig. 11B and Fig 11C. Since the structure is stable with the atomistic (both effective Gaussian model and explicit solvent), as well as with the coarse-grained representation provides evidence that the results are reliable, despite variations in the model representation. The coarse-grained model may be applied to studying the assembly process of unit length filaments or the compacting process of full length filaments.

Our analysis illustrates the opportunities associated with a computational approach in describing complex biological materials. The study reported here provides a new way forward in carrying out molecular-level studies of IF structure-property relationships. This may be particularly important to probe the effects of the variability of the structure and associated changes in chemical and intermolecular interactions (e.g. amino acid mutations and other molecular defects). Defects may be introduced at different hierarchical levels of the structure of vimentin intermediate filament, and the effects on the biomechanical performance may provide important insight into physiological and diseased states of IFs. This specifically includes the possibility of probing the effect of mutations using *in silico* materiomics methods, enabling pathogenesis studies of severe medical conditions associated with IF. The model sets the stage for studies ranging from *in vivo* cross-linking events (e.g. how keratinocyte transglutaminase crosslinks keratins in the skin) to investigating the effects of protein binding on IF mechanics. Due to the emerging significance of intermediate filaments in a variety of cellular process, the results of our study also contribute to improved understanding of cell deformation, cell adhesion and mechanotransduction. IFs can also be considered a model system that may enable us to fabricate *de novo* engineered materials that display a high sensitivity to applied forces, show resilient mechanical properties, and provide biological compatibility. Another possible next challenge of using such models is to apply them to describe larger-scale cellular structures [47] and to develop bottom-up models of dynamic processes such as cell signaling and protein expression. For example, IF networks *in vivo* are in equilibrium with (and interact with) a pool of soluble IF associated proteins that are able to exchange anywhere along the filaments. With our approach, we may be able to address how moderate tension may change the exchange rate.

The concept of designing materials with hierarchical structures, by deliberately determining a cascade of multi-scale mechanisms as shown in Table 1 is a largely unexplored aspect in materials science that could lead to advances in *de novo* materials design. By utilizing self-assembly processes from nano to macro, hierarchical structures may be the key that can enable us to take advantage of properties at all scales, and to exploit superior nanoscale properties. This could lead to a new class of biocompatible natural materials with applications as stimulus responsive gels, self-assembled nanomaterials, biomaterials, or as energy absorbing materials.

## Materials and Methods

### Atomistic level molecular models and force fields

Molecular simulations are carried out using the CHARMM19 all-atom energy function with an effective Gaussian model for the water solvent [22], [23]. Additional simulations are carried out using explicit solvent models with the CHARMM force field and explicit TIP3 water [24] implemented in NAMD [25] for validation of the effective Gaussian simulations. In both models, we apply a 1 femtosecond time step, with constant temperature (300 K). A pressure of 1 atmosphere is used in the explicit water simulations.

The implicit water molecular model is currently the only feasible approach to simulate sufficiently large protein structures at the atomistic level at long time-scales (reaching tens of nanoseconds), and is therefore used for structure prediction and mechanical characterization. The explicit solvent model and the coarse-grained approach is used to validate the results of implicit solvent simulations. The use of the effective solvent model is further motivated by our desire to overcome the limitations of relatively fast unfolding rates used in the simulations. The lack of slow water relaxation processes in the implicit model enables us to effectively model the physical conditions of pulling experiments at much longer time-scales. Therefore, effective solvent methods provide physically more meaningful results than the use of explicit solvent methods, as discussed in [48], [49], [50].

Several other studies have recently utilized the approach used here. For example, the effective Gaussian model for the water solvent was used in mechanical unfolding studies of proteins in [48] and [50], where mechanical properties of several types of protein segments were studied. Structure prediction approaches similar as used here have also been reported in [49], where the conformation of collagen structures has been studied. Due to the size of the protein, the simulations reported here take up to 50 days each on a parallel Linux cluster.

### Coarse-grained model and force field

In addition to atomistic level simulations we carry out an equilibration of the molecular structure by using the coarse-grained MARTINI force field [26] in explicit water., to explore the structural stability of the predicted structures at time-scales of several hundred nanoseconds. The initial coarse-grained geometry (shown in Fig. 11A) is generated from the full atomistic model of the tetramer (after the structure prediction process). We use a 40 femtosecond time step in this coarse-grained model, and the same constant temperature and pressure as in the full atomistic model. The coarse-grained system includes 43,801 particles (4,052 for the protein plus 39,749 for water molecules), while the corresponding full atomistic system would include 506,915 atoms (29,924 atoms for the protein plus 476,991 atoms for water molecules). The number of particles in the coarse-grained model is 1/11 of the full atomistic representation.

### Molecular model setup

The structure of the dimer is predicted by a series of computational steps as summarized in Fig. S1. First, the consideration of experimental sequence information and associated structural features enables us to develop an initial configuration that is close to the naturally favored state. Through this approach, we avoid solving a complete protein folding problem and rather focus on structural optimization close to the equilibrium geometry. Alpha-helical parts are bundled and rotated considering the orientation of their hydrophobic strikes. Based on the initial structure, equilibrated geometries are obtained through a series of repeated structural optimization steps, using both energy minimization and equilibration (following an approach suggested in [22], [23]). Figure S2 shows the evolution of the total energy and the root mean square deviation (RMSD) for both structures, during the last 10 ns of the equilibration period. The convergence of the two curves at constant values suggests that both the dimer and tetramer have reached a stable configuration.

### Validation of molecular model

To confirm the stability of the molecular structures, we carry out simulations in explicit solvent using the CHARMM force field with explicit solvent [24]. Figures S3 and S4 show the results of radial distribution function analyses, comparing the structures in implicit and explicit solvent for both the dimer and tetramer, confirming the structural stability of the molecular models. Furthermore, we compare the radial distribution function of our model with the existing segments obtained from x-ray diffraction (PDB entries 1gk7 for the 1A segment and 1gk4 for the 2B segment), showing good agreement as depicted in Fig. S5.

The atomistic geometry resulting from the structure prediction runs is also used to build a coarse-grained representation of the vimentin tetramer using the MARTINI force field [26], where an analysis of the stability of the molecule at hundreds of ns time-scales (Figure 11) and the analysis of the radial distribution function of the backbone particles (Fig. S6) suggests that the predicted geometry is stable.

Altogether, these comparisons and the structural data shown in Figure 3 and Table S1 suggests that the molecular models of the dimer and tetramer are reasonable representations of the vimentin IF dimer and tetramer structures.

### Nanomechanical characterization

The 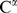 atoms at the end of two 2B segments are pulled on by using steered molecular dynamics, while the other end of the filament is fixed (with a force constant of 10 kcal/mol/Å^2^). The pulling force *F* is recorded versus the position. The simulations are carried out at pulling velocities ranging from 0.0001 Å/ps to 1 Å/ps. It is noted that 0.1 Å/ps is the lowest computationally accessible pulling speed for the larger tetramer system in which we can perform stretching until failure occurs. The analysis of the mechanical properties is facilitated by calculating engineering stress and strain. We record the force-displacement curve from steered molecular dynamics simulation and analyze the mechanical properties by computing the engineering stress and strain, which are defined as
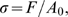
(1)and
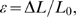
(2)where 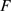, 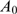, 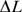 and 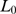 are the pulling force, relevant cross-sectional area, displacement, and initial length, respectively. The (tangent) Young's modulus is determined by
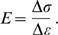
(3)


### Comparison between full-length filament experiment and simulations

The comparison between simulations of tetramers and experiment results of full length IFs as shown in Fig. 4C is done as follows. In full length filaments there are no free ends. Therefore the structure is regarded as a rolled sheet, which is composed of eight overlapping parts. To reflect the lack of free ends in full length filaments we remove the part of the force-strain data that corresponds to the initial unfolding of the free ends. The modified strain corresponding solely to the overlapped part is obtained by converting the strain in the third and forth region in Fig. 4B following
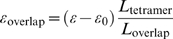
(4)(where 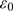 is the beginning of the third region). We shift the simulation force to begin from zero at 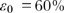  = 60%. To compute the engineering stress, we divide the force by the cross-sectional area of the tetramer, 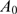  = 32.9 nm^2^ (for details of geometric analysis, please see Supporting Methods S1). Under changes in strain rate, the strength of protein filaments increase by a factor compared with the quasi-static loading observed in experiment [35], [51]. To account for this effect, we calculate a force scaling factor to extrapolate the simulation results to the strain rate used in experiment, specifically considering the rate dependence of beta-sheet proteins (since the failure of beta-sheets accounts for the final peak in the stress-strain curve). Earlier studies have revealed that for beta-sheet proteins (under shear loading) the strength at 0.1 Å/ps pulling speed is 1,700 pN [52]. For slow pulling on the order of 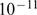 Å/ps the strength is found to be 240 pN [53]. This leads to a scaling factor value of 7.1 to account for the different strain rates in experiment and simulation. This facilitates a direct comparison with experimental results using AFM [8].

### Molecular structure analysis

We use Visual Molecular Dynamics (VMD) [54] for visualization of protein structures as well as for the analysis of the *α-β* transition shown in Figures 8 and 9. The structure analysis is done using the approach suggested in [55]. The rupture length of H-bonds is 3.7 Å for visualization in VMD. The criterion to identify H-bonding considers both hydrogen bond patterns and the backbone geometry, based on polypeptide chain dihedral angles.

## Supporting Information

Methods S1 Additional information about computational methods, data analysis and research approach.
(0.10 MB PDF)

Table S1 Structural parameters of the dimer/tetramer structure and a quantitative comparison between experiment and simulation. Part of the data shown here is visualized in Figure 3.
(0.01 MB PDF)

Table S2 Mechanical properties of vimentin IF, quantitative comparison between experiment and simulation. Part of the data shown here is visualized in Figure 5.
(0.06 MB PDF)

Figure S1 Overview over the structure prediction approach used here. The incorporation of structural features based on amino acid sequence is used to create an initial structural model. A sequence of energy minimization and equilibration, repeated until convergence is reached, results in the final structure that is validated against experimental results and then used for mechanical analysis.
(0.03 MB PDF)

Figure S2 Total energy and root mean square displacement (RMSD) analysis for the last 10 ns of the equilibration process, for the dimer (panel A) and the tetramer (panel B).
(0.03 MB PDF)

Figure S3 Radial distribution function (RDF) for both models in implicit solvent and explicit solvent (panel A: dimer, panel B: tetramer). The peaks represent the distance from an alpha-carbon atom to the nearest neighbor alpha-carbon atoms, indicating the secondary and tertiary structure of coiled-coil proteins. The same location of the peaks means that structural characters are same for our protein model in both the implicit solvent and explicit solvent environment.
(0.03 MB PDF)

Figure S4 Integrated of RDF function for both models in implicit solvent and explicit solvent (panel A: dimer; panel B: tetramer).
(0.03 MB PDF)

Figure S5 Comparison of RDF analysis between our model and experimental results (based on the model obtained through x-ray diffraction analyses), for the 1A segment (panel A), and for the 2B segment (panel B). The peaks represent the distance from an alpha-carbon atom to the nearest neighbor alpha-carbon atoms, indicating the secondary and tertiary structure of coiled-coil proteins. The same location of the peaks means that structural characters are same for our protein model and experimental model.
(0.03 MB PDF)

Figure S6 Comparison of the RDF between the full-atomistic model and the coarse-grained representation, after 300 ns equilibration. The peaks represent the distances from a backbone bead to the nearest neighbor backbone beads, indicating the secondary and tertiary structure of coiled-coil proteins. The same location of the peaks means that structural characters are same for our protein model in both the implicit solvent and explicit solvent environment.
(0.04 MB PDF)

Structure S1 Atomistic structure of the intermediate filament dimer in the Protein Data Bank (PDB) format.
(0.74 MB TXT)

Structure S2 Atomistic structure of the intermediate filament tetramer in the Protein Data Bank (PDB) format.
(1.47 MB TXT)

Movie S1 Equilibrated structure of the vimentin IF dimer at 300 K. The movie shows a 5 ns interval of a constant temperature simulation of the dimer in water solvent.
(6.79 MB AVI)

Movie S2 Equilibrated structure of the vimentin IF tetramer at 300 K. The movie shows a 5 ns interval of a constant temperature simulation of the dimer in water solvent.
(8.36 MB AVI)
