# Supplementary material for: Hierarchical Structure Controls Nanomechanical Properties of Vimentin Intermediate Filaments
Source: PLoS One. 2009 Oct 6;4(10):e7294. doi: 10.1371/journal.pone.0007294 (PMC2752800; doi:10.1371/journal.pone.0007294)
Supplement: Methods S1 — Additional information about computational methods, data analysis and research approach. (0.10 MB PDF) [file pone.0007294.s001.pdf]

Supporting Methods S1

Molecular model structure identification

The structure identification simulations are carried out using the CHARMM program by using the CHARMM19 all-atom energy function and an implicit Gaussian model for the water solvent [22,23]. The use of the implicit solvent model facilitates a more rapid structural organization towards the equilibrium geometry due to the lack of viscosity imposed by explicit water molecules. This effect is advantageous here since it accelerates the sampling of molecular configurations, so that more configurations are visited per simulated nanosecond. Overall, the use of this solvation potential has been shown to be an efficient and reliable approach to take into account solvent effects of large molecular structures suitable for structure prediction applications [48,56,57]. This molecular model is currently the only feasible approach to simulate large protein structures as considered here at sufficiently long time scales required for structural equilibration (due to the size of the protein, the simulations for the tetramer structure reported here take up to 50 days on a parallel cluster). Additional explicit solvent simulations [24] are used to validate the stability of the equilibrated structures, showing that the structures predicted by our approach are stable also in explicit solvent. The time step used in all simulations is 1 fs.

Generation of initial geometry of dimer and tetramer

The key for the structure optimization protocol used here to work is the generation of an initial geometry that is reasonably close to the naturally favored state, in order to be able to simulate the structural rearrangements within the time-scale accessible to molecular dynamics simulations. Through this approach, we avoid solving a complete protein folding problem and rather focus on structural optimization close to the equilibrium geometry.

In order to obtain a good initial structure, we set up the initial atomistic model by considering the amino acid sequence and associated structural information obtained from x-ray diffraction (XRD) experiments and other experimental approaches [15,16,17,58]. The initial structural model constructed based on this approach is an appropriate starting configuration because all

proteins with more than 50% sequence identity have been shown to feature identical folds [59]. Once the initial geometry is set up, no constraint is applied during the energy minimization and equilibration phases of the simulation. This implies that the molecular structure is free to rearrange and restructure in any possible configuration.

Each polypeptide chain of the dimer has the identical 466 amino acid sequence as to reflect the exact sequence of human vimentin intermediate filaments [16]. The amino acids are connected by taking $\phi = -58^\circ$ and $\psi = -47^\circ$ for the known conformation of alpha-helical segments [60], and by taking $\phi = 180^\circ$ and $\psi = 180^\circ$ for the other unstructured parts of the chain. Note that the parameters ϕ and ψ are two characteristic dihedral angles that define the conformation of the protein backbone chain; these angles describe how two neighboring amino acids are connected to one another (for details see [61]). According to the sequence analysis, the 1A, 1B, 2A and 2B segments possess an initial alpha-helical structure, while the other parts of the chain are initially unstructured [14,15,16].

Each amino acid within the polypeptide chain takes the topology and local coordinates according to the optimized geometry in the CHARMM 22 force field [24]. Due to the polarities of the amino acids, the apolar amino acids in the 1A, 1B and 2B segments generate a hydrophobic stripe that winds around its helical axis with a left-handed heptad repeat. Via coordinate displacements and transformations, we combine two initially straight alpha-helical chains together with specific distance and coiled angle to form a coiled-coil structure. This configuration is energetically favored since it shields apolar residues from the polar solvent environment [58]. The coiling angle is 3.51 deg/Å for 1A, 3.85 deg/Å for the 1B and 2.46 deg/Å for the 2B segment. The coiling angles are different among different segment because the twist angle of the hydrophobic stripe on each of the segments differs. This twist angle is determined by linear fitting of the angular coordinate θ of the hydrophobic amino acids with respect to their axial coordinate z in the $R-\theta-z$ coordinate system of C_α atoms. In the 2A segment, the hydrophobic stripe is nearly parallel to the axis of the alpha-helix, resulting in a parallel alpha-helical bundle. The initial tetramer model is created by placing two dimer models in an

antiparallel stagger.

Overall structure prediction approach

The structure of the dimer is predicted by a series of computational steps as summarized in Fig. S1. Starting from the initial geometry as obtained by the method outlined above we follow the following protocol to equilibrate the structure:

1. Energy minimization (10,000 Steepest Descent steps followed by 10,000 Adopted Basis Newton-Raphson steps).

2. Equilibration run for 5 ns (NVT dynamics with Nose-Hoover temperature control), where the temperature rises linearly from 240 K (beginning) to 300 K (end) (following the same approach as used in [62]).

Steps 1 and 2 are repeated 10 times to ensure convergence. A final equilibration run at 300 K for 10 ns is completed at the end of the cycle. The repeated process of energy minimization and equilibration ensures the convergence towards the equilibrated structure. Aside from random fluctuations of the molecule, no change in the molecular structure is observed after a few iterations, suggesting that the ten cycles are sufficient to reach a properly equilibrated structure.

Validation of structure identification results in explicit solvent

To confirm the structure predictions, we carry out explicit solvent simulations with the CHARMM force field and explicit TIP3 water [24] implemented in NAMD [25], starting from the equilibrated structure obtained using the approach described above. Both models are of substantial size and contain more than 500,000 atoms. A total time interval of 10 ns is computed for both the dimer and tetramer. By following the RMSD over time we confirm that the predicted structures of the dimer and the tetramer are both stable and that they show no major structural changes.

Furthermore, we carry out an analysis of the radial distribution function (RDF) to determine

local and medium range structural features and their comparison between the implicit and explicit solvent models. The RDFs are calculated using the "gofr" plug-in in VMD. It is noted that our analysis approach is the same as in reference [63]. The RDFs are obtained by averaging over the last 100 ps of the equilibration runs in both cases. The results shown in Figure S3 confirm that in both the two figures, the RDF peaks for implicit solvent is the same as that for the explicit solvent, which means the structure for the protein within two solvent features the same character. The first peak at 3.84 A is related to the character of the primary structure. The other four peaks for 5.00 Å, 5.46 Å, 6.15 Å, and 8.70 Å, respectively, denote the first, second, third and forth neighbors in the secondary and tertiary structures of the protein filament, respectively. Furthermore, the structural stability can be clearly seen if we integrate the RDF by $f(x) = \int_0^x g(r)dr$. The results of this analysis are shown in Figure S4.

Figure S5 displays a comparison of the RDF between structures obtained from x-ray diffraction and our molecular model (the existing crystallized segment for vimentin is 1GK7 for the 1A segment, and 1GK4 for the 2B segment). Both structures show good agreement between simulation and experiment.

Validation of structure identification results using the coarse-grained model

Our coarse-grained model is a residue based coarse-grained representation. The initial geometry is generated from the full atomistic model (as described above). The model for the tetramer includes 43,801 particles (4,052 for the protein plus 39,749 for the water molecules), while the corresponding full atomistic system includes 506,915 particles (29,924 for the protein plus 476,991 for the water molecules). Fig. 11A shows the full view of the coarse-grain model as well as a comparison with the full atomistic one. Each amino-acid is coarse-grained into 1 to 5 particles. The initial coordinates are generated from the atomistic system.

Using this model, we carry out an equilibration for 300 ns = 0.3 μs. We use a time step of 40 fs, and keep the system temperature at 300 K, the pressure at 1 atmosphere (reflecting the same condition as for our full atomistic system). The RMSD analysis shown in Fig. 11B suggests that the tetramer structure has reached an equilibrated state after ≈150 ns. Most importantly, there is

no conformational change during the equilibration process. This is also confirmed by the RDF analysis as shown in Fig. S6. The RDF peaks for the backbone particles in the initial coarse-grained model remain the same as that of the model at equilibrium, which means the structural character of the coarse-grained tetramer remains constant.

Geometrical analysis

The equilibrated structures of the dimer and tetramer are subjected to geometrical analysis. The cross-section of the dimer is calculated by

$$A_0^{\text{dimer}} = \frac{1}{4}\pi d_{\text{dimer}}^2, \tag{S1}$$

where d_{dimer} is the average diameter of the dimer structure.

For the tetramer, the calculation is more complex. By taking into consideration that eight tetramers are arranged in a circle in the non-compacted unit length filament as experimentally determined in [15], the unit length filament diameter is $d_{\text{ULF}} = 16 d_{\text{tetramer}} / \pi$. Taking the IF to be a solid cylinder as suggested in [15], the cross-sectional area for the non-compacted unit length filament is given by:

$$A_0^{\text{ULF}} = \frac{1}{4}\pi d_{\text{ULF}}^2. \tag{S2}$$

For the tetramer, we consider the IF as a solid cylinder to calculate the cross-sectional area, as done in corresponding experimental studies [15,34]. Therefore, the cross-sectional area for the tetramer in the non-compacted state is calculated from A_0^{ULF} to be

$$A_0^{\text{tetramer}} = A_0^{\text{ULF}} / 8. \tag{S3}$$

The cross-sectional area of mature, compacted IFs can be regarded as a closed-packed arrangement of eight tetramers. Assuming that each tetramer in the compacted state features a

circular cross-sectional area of $A_0^{\text{tetramer,C}} = \pi / 4 d_{\text{tetramer}}^2$, the total cross-sectional area of a mature, compacted full length filament is:

$$A_0^{\text{FLF,C}} = 8\alpha A_0^{\text{tetramer,C}} \qquad \text{(S4)}$$

where the factor of α =1.10 accounts for additional free space in the hexagonal packing of tetramers. Considering that mature full length filaments feature a circular cross-section, the resulting effective diameter is given by

$$d_{\text{IF, mature}} = \sqrt{4 A_0^{\text{FLF,C}} / \pi}\ . \qquad \text{(S5)}$$

Supporting references

56. Best RB, Merchant KA, Gopich IV, Schuler B, Bax A, et al. (2007) Effect of flexibility and cis residues in single-molecule FRET studies of polyproline. Proc Natl Acad Sci U S A 104: 18964-18969.
57. Paci E, Karplus M (1999) Forced unfolding of fibronectin type 3 modules: An analysis by biased molecular dynamics simulations. Journal of Molecular Biology 288: 441-459.
58. Apgar JR, Gutwin KN, Keating AE (2008) Predicting helix orientation for coiled-coil dimers. Proteins-Structure Function and Bioinformatics 72: 1048-1065.
59. Dalal S, Balasubramanian S, Regan L (1997) Protein alchemy: Changing beta-sheet into alpha-helix. Nature Structural Biology 4: 548-552.
60. Brändén C-I, Tooze J (1999) Introduction to protein structure. New York: Garland Pub. xiv, 410 p. p.
61. Alberts B, Johnson A, Lewis J, Raff M, Roberts K, et al. (2002) Molecular Biology of the Cell. New York: Taylor & Francis.
62. Miteva MA, Brugge JM, Rosing J, Nicolaes GA, Villoutreix BO (2004) Theoretical and experimental study of the D2194G mutation in the C2 domain of coagulation factor V. Biophys J 86: 488-498.
63. Jones MK, Catte A, Patterson JC, Gu F, Chen J, et al. (2009) Thermal stability of apolipoprotein A-I in high-density lipoproteins by molecular dynamics. Biophys J 96: 354-371.
