# Supplementary material for: Hierarchical Structure Controls Nanomechanical Properties of Vimentin Intermediate Filaments
Source: PLoS One. 2009 Oct 6;4(10):e7294. doi: 10.1371/journal.pone.0007294 (PMC2752800; doi:10.1371/journal.pone.0007294)
Supplement: Table S1 — Structural parameters of the dimer/tetramer structure and a quantitative comparison between experiment and simulation. Part of the data shown here is visualized in Figure 3. (0.01 MB PDF) [file pone.0007294.s002.pdf]

Table S1: Structural parameters of the dimer/tetramer structure and a quantitative comparison between experiment and simulation. Part of the data shown here is visualized in Figure 3.

**Geometrical feature**	**Experimental**	**Simulation (this study)**
L1 domain	Unstructured [14,17]; Suggested that it facilitates assembly [32]	Unstructured (see Fig. 2B, blow-up); Facilitates assembly via alignment with L12 in tetramer (see Figure 2C, blow-up)
L12 domain	Beta-sheet structure [32]	Beta-sheet structure in dimer (see Fig. 2B, center blow-up) ;Facilitates assembly via alignment with L2 in tetramer (see Figure 2C, blow-up)
L2 domain	Unstructured [14,17];	Unstructured (see Fig. 2B, blow-up)
Segment 2A	Extended parallel alpha-helices [14]	Extended parallel alpha-helices
Structure in vicinity of residue 349	Stutter (=locally parallel alpha-helices) [16,30]	Stutter (see Fig. 2B, circled region)
Dimer length	46-49 nm [15,27]	48.7 nm
Unit length filament length	62 nm [27]	61.3 nm
Overlap part length in tetramer	30-36 nm [15,27]	36 nm
Dimer diameter, d_{dimer}	2-3 nm [15]	2 nm
Tetramer diameter, d_{tetramer}	3.4 nm [15]	3.6 nm
Diameter of a non-compacted unit length filament, d_{ULF}	16-20 nm [33]	18.3 nm
Compacted intermediate filament diameter, $d_{\text{IF, mature}}$	10-12 nm [33,34]	11.2 nm
