# Supplementary material for: Hierarchical Structure Controls Nanomechanical Properties of Vimentin Intermediate Filaments
Source: PLoS One. 2009 Oct 6;4(10):e7294. doi: 10.1371/journal.pone.0007294 (PMC2752800; doi:10.1371/journal.pone.0007294)
Supplement: Table S2 — Mechanical properties of vimentin IF, quantitative comparison between experiment and simulation. Part of the data shown here is visualized in Figure 5. (0.06 MB PDF) [file pone.0007294.s003.pdf]

Table S2: Mechanical properties of vimentin IF, quantitative comparison between experiment and simulation. Part of the data shown here is visualized in Figure 5.

**Mechanical property**	**Experimental**	**Simulation (this study)**
Overall stress-strain behavior	Soft for small strains, stiffening at large strains (found in [8,34])	Soft for small strains, stiffening at large strains (Fig. 4C)
Small-strain modulus	300-900 MPa [38]	380-540 MPa
Tangent modulus at ≈50% strain	7..15 MPa [8,34]	3..12 MPa
Stiffening onset	150..180% [8]	100% (gradual)
Tangent modulus at ≈150%	105 MPa	164 MPa
Tangent modulus before failure	290 MPa	185 MPa
Yield strain	180% [8]	175%
Stiffness at yield	Softening (from 180% to 240%) [8]	Softening (from 175% to 300%)
Yield stress	90 MPa [8]	97 MPa
Average yield stress	90 MPa [8]	79 MPa
Failure strain	240-300%	300%
Filament diameter change after yield	Thinning (intermolecular sliding suggested) [43]	Thinning due to intermolecular sliding (Fig. 10)
Structural transition at large deformation	Beta-sheet formation (α-β transition) [34,41,42]	Beta-sheet formation (α-β transition; Figs. 8 and 9)
