# Supplementary material for: Hierarchical Structure Controls Nanomechanical Properties of Vimentin Intermediate Filaments
Source: PLoS One. 2009 Oct 6;4(10):e7294. doi: 10.1371/journal.pone.0007294 (PMC2752800; doi:10.1371/journal.pone.0007294)
Supplement: Figure S1 — Overview over the structure prediction approach used here. The incorporation of structural features based on amino acid sequence is used to create an initial structural model. A sequence of energy minimization and equilibration, repeated until convergence is reached, results in the final structure that is validated against experimental results and then used for mechanical analysis. (0.03 MB PDF) [file pone.0007294.s004.pdf]

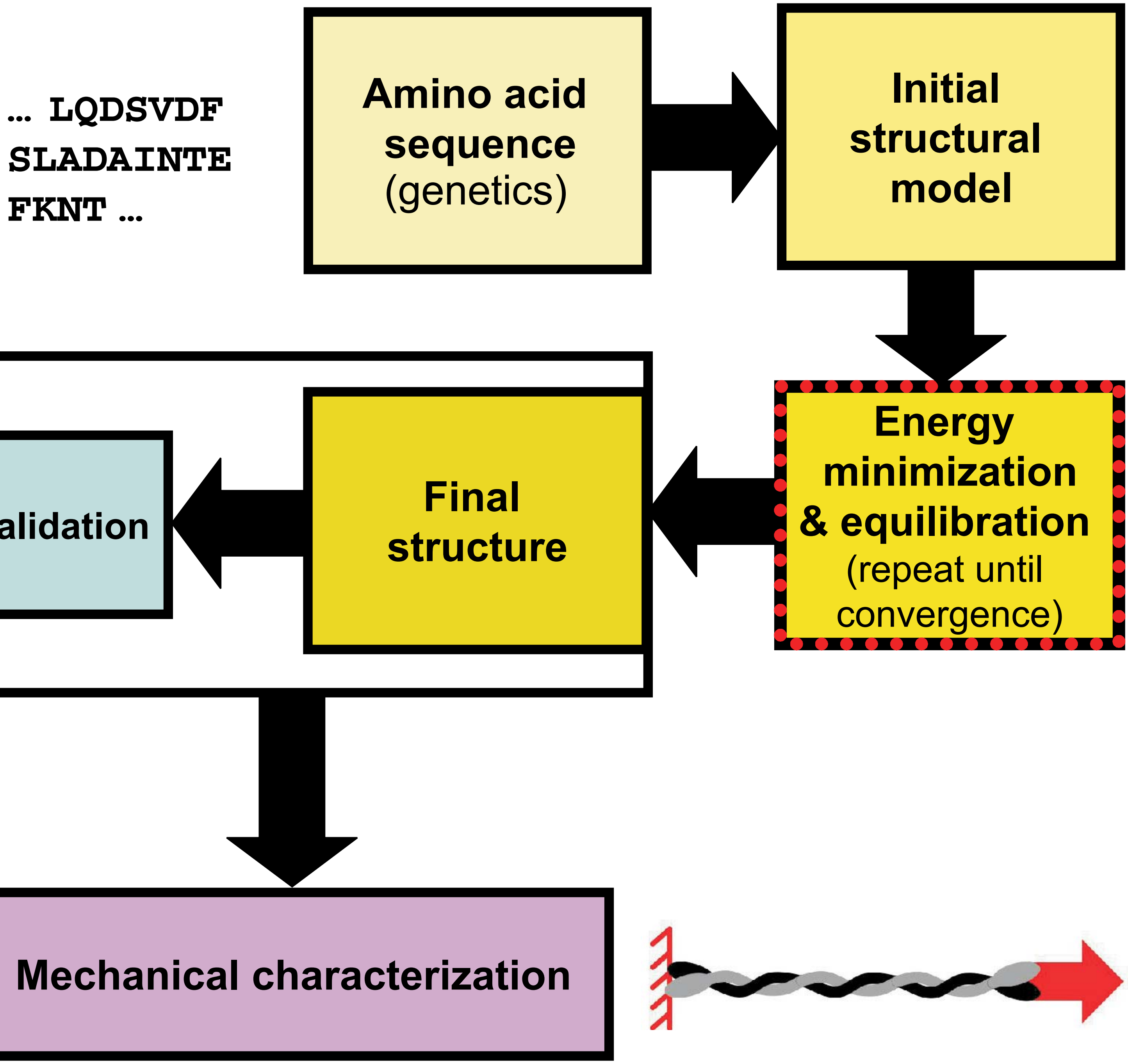
… LQDSVDF
SLADAINTE
FKNT …
Amino acid
sequence
(genetics)
Initial
structural
model
Energy
minimization
& equilibration
(repeat until
convergence)
Final
structure
Validation
Mechanical characterization
