# Supplementary figures and images for: Hierarchical Structure Controls Nanomechanical Properties of Vimentin Intermediate Filaments
Source: PLoS One. 2009 Oct 6;4(10):e7294. doi: 10.1371/journal.pone.0007294 (PMC2752800; doi:10.1371/journal.pone.0007294)

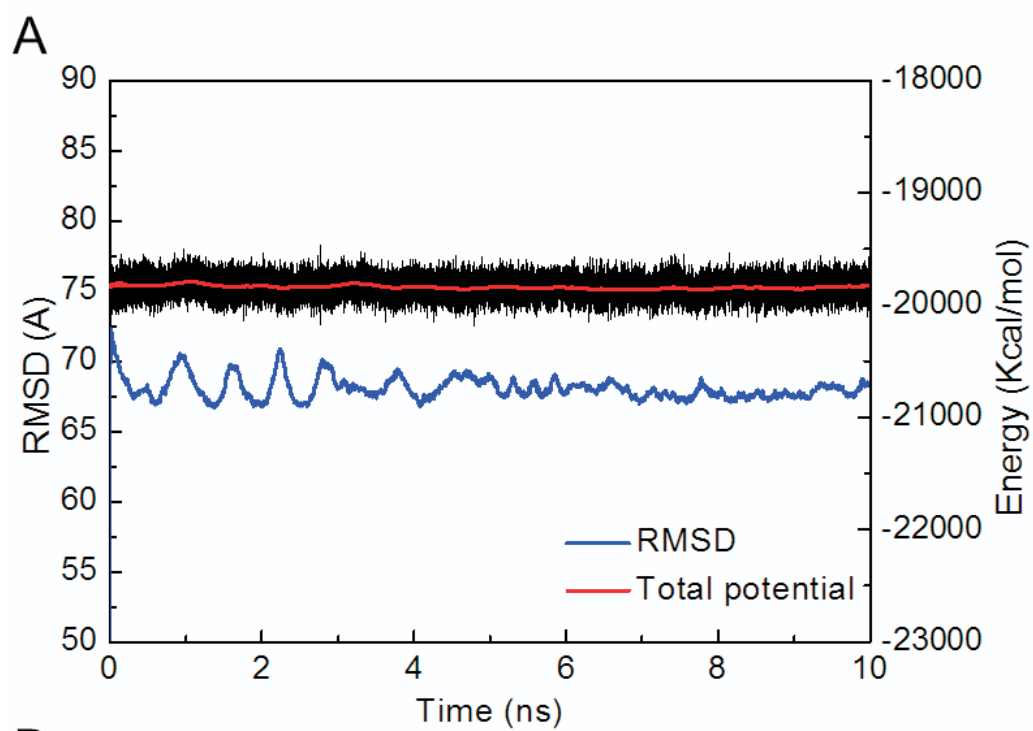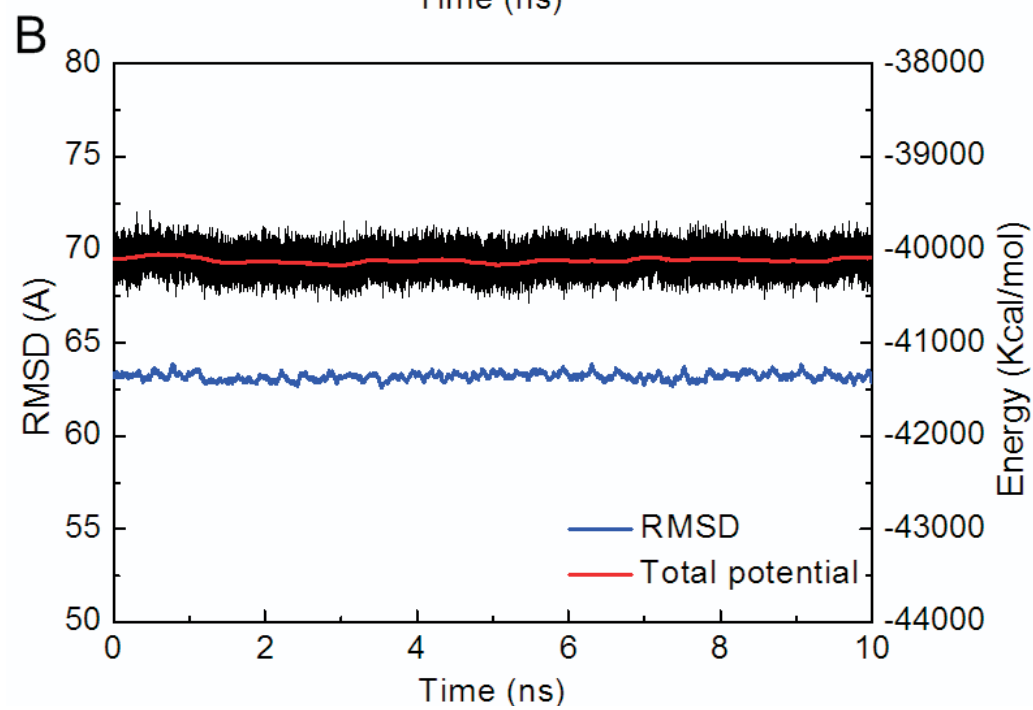

Supplement: Figure S2 — Total energy and root mean square displacement (RMSD) analysis for the last 10 ns of the equilibration process, for the dimer (panel A) and the tetramer (panel B). (0.03 MB PDF) [file pone.0007294.s005.pdf]

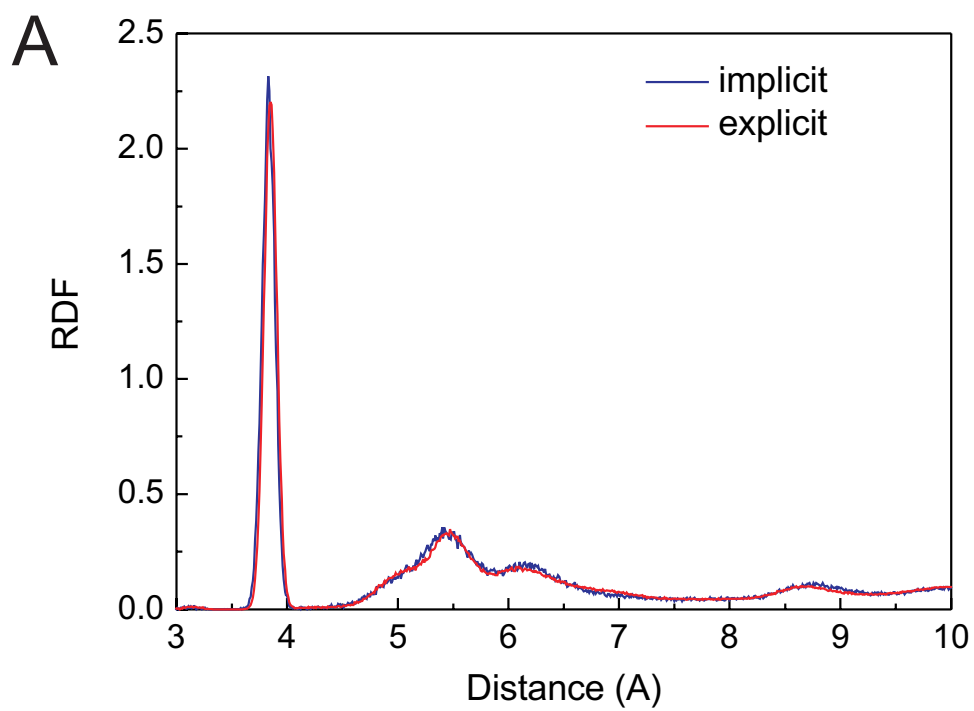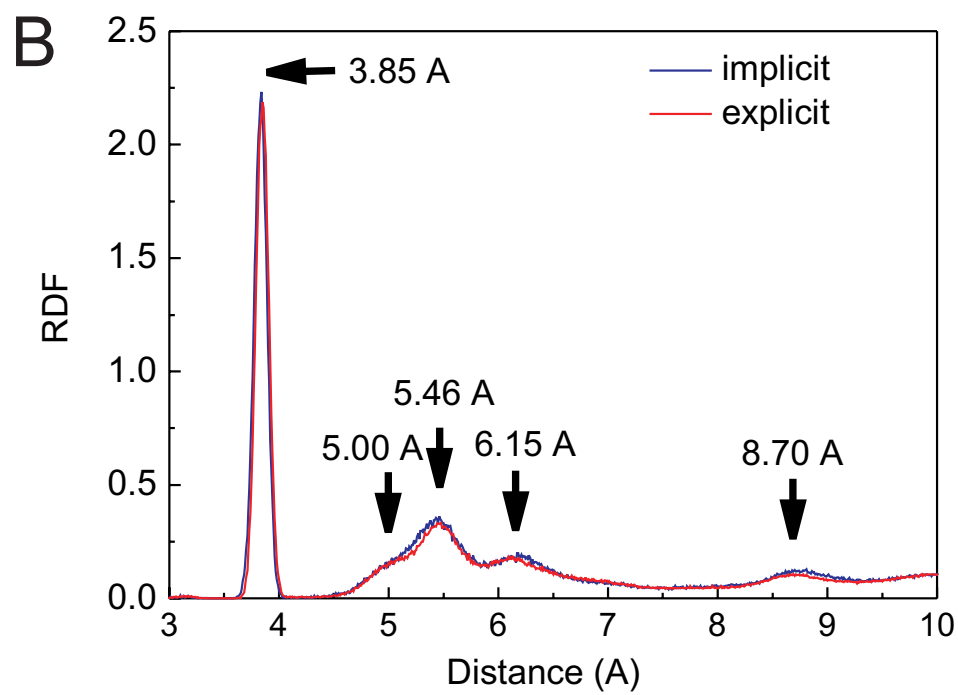

Supplement: Figure S3 — Radial distribution function (RDF) for both models in implicit solvent and explicit solvent (panel A: dimer, panel B: tetramer). The peaks represent the distance from an alpha-carbon atom to the nearest neighbor alpha-carbon atoms, indicating the secondary and tertiary structure of coiled-coil proteins. The same location of the peaks means that structural characters are same for our protein model in both the implicit solvent and explicit solvent environment. (0.03 MB PDF) [file pone.0007294.s006.pdf]

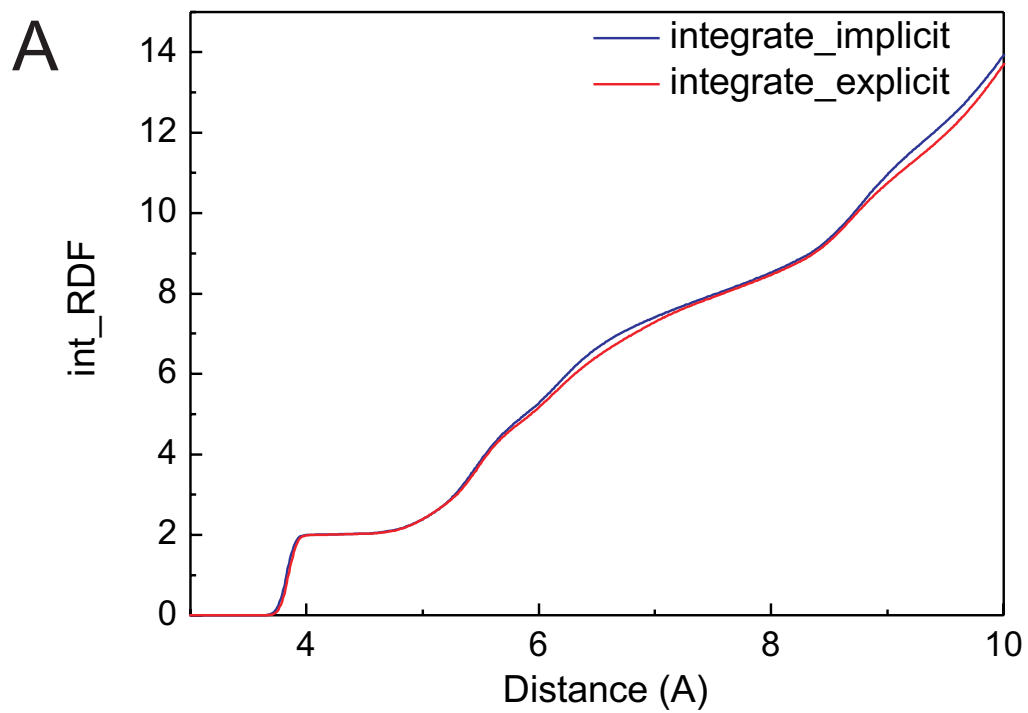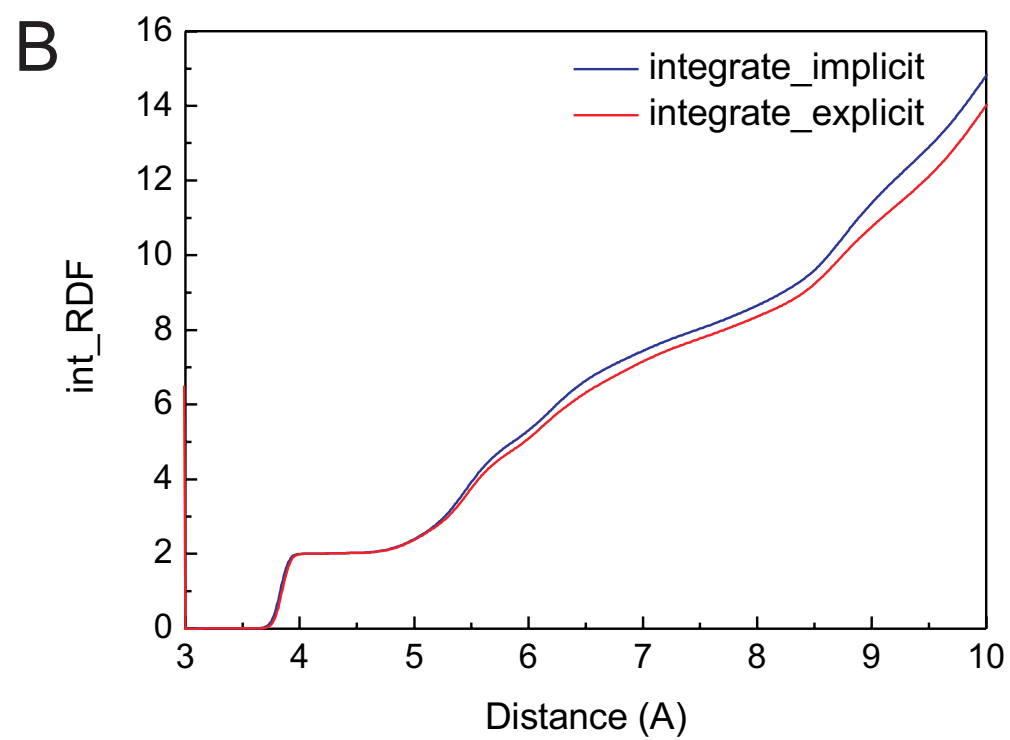

Supplement: Figure S4 — Integrated of RDF function for both models in implicit solvent and explicit solvent (panel A: dimer; panel B: tetramer). (0.03 MB PDF) [file pone.0007294.s007.pdf]

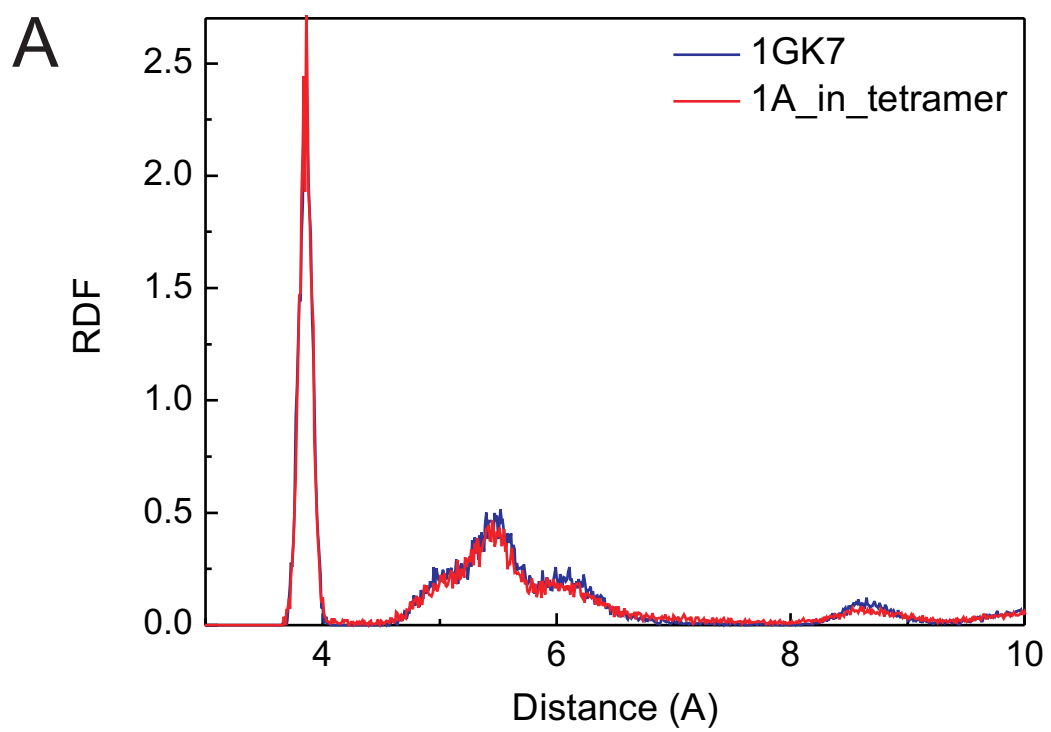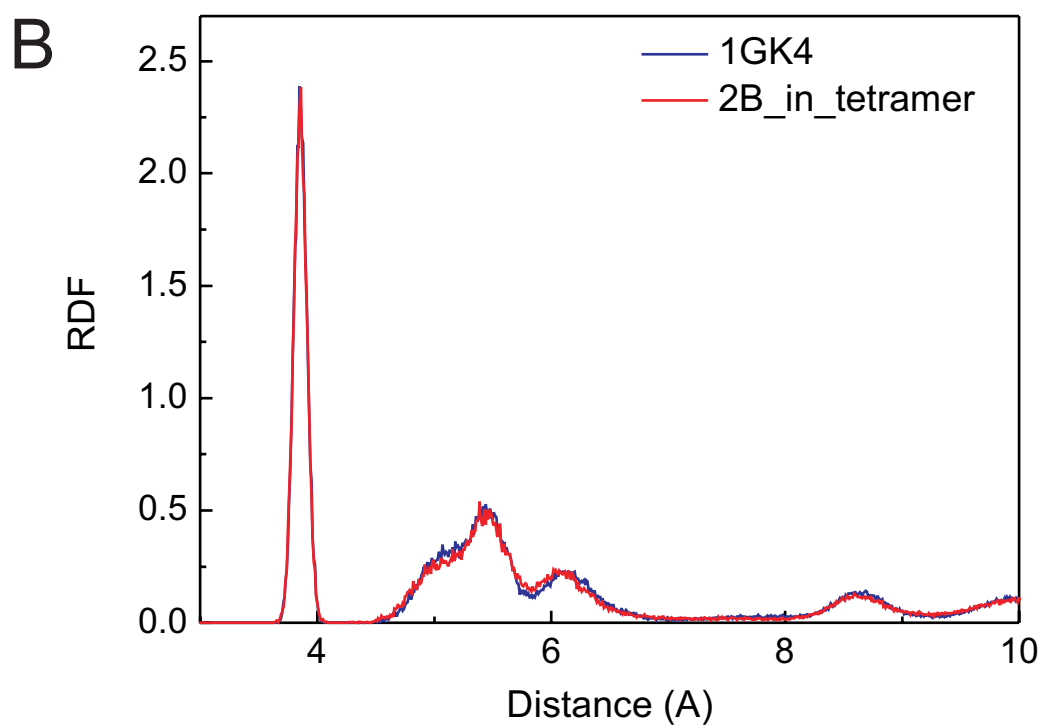

Supplement: Figure S5 — Comparison of RDF analysis between our model and experimental results (based on the model obtained through x-ray diffraction analyses), for the 1A segment (panel A), and for the 2B segment (panel B). The peaks represent the distance from an alpha-carbon atom to the nearest neighbor alpha-carbon atoms, indicating the secondary and tertiary structure of coiled-coil proteins. The same location of the peaks means that structural characters are same for our protein model and experimental model. (0.03 MB PDF) [file pone.0007294.s008.pdf]

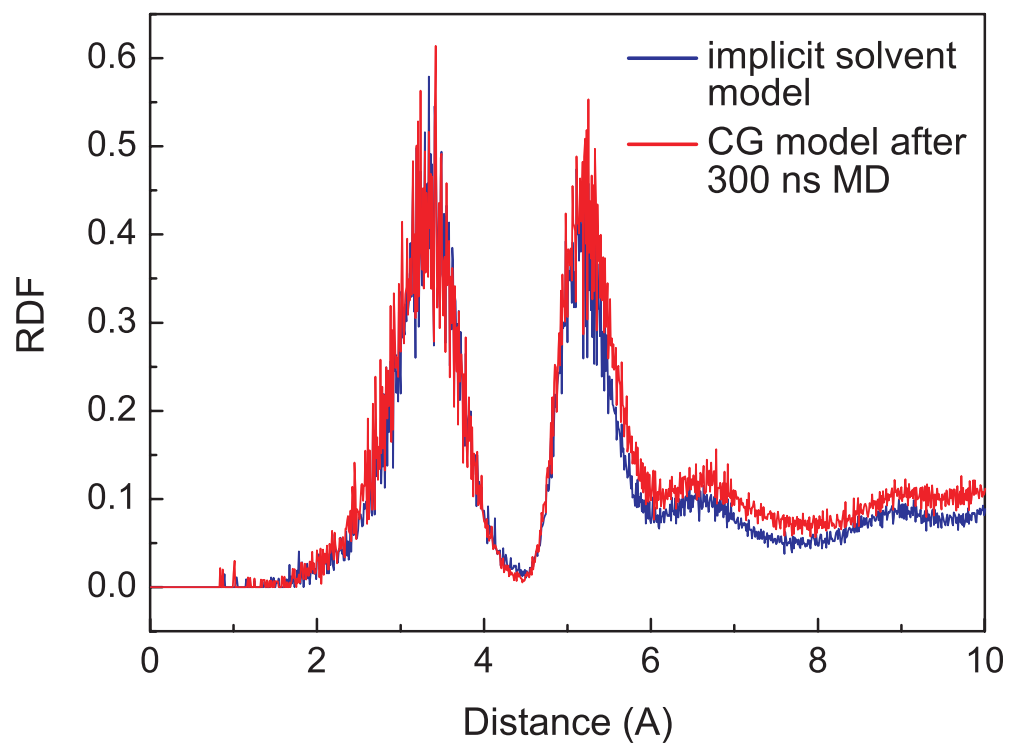

Supplement: Figure S6 — Comparison of the RDF between the full-atomistic model and the coarse-grained representation, after 300 ns equilibration. The peaks represent the distances from a backbone bead to the nearest neighbor backbone beads, indicating the secondary and tertiary structure of coiled-coil proteins. The same location of the peaks means that structural characters are same for our protein model in both the implicit solvent and explicit solvent environment. (0.04 MB PDF) [file pone.0007294.s009.pdf]
